# A Step-by-Step Approach to a Successful Cosmetic Breast Reduction

**DOI:** 10.1097/GOX.0000000000002117

**Published:** 2019-04-02

**Authors:** Rodrigo Guridi, José Ramón Rodriguez

**Affiliations:** From the Department of Plastic and Reconstructive Surgery, Clínica Las Condes, Santiago, Chile.

## Abstract

Evolution of breast reduction techniques has been a result of combining different skin marking patterns with a wide variety of pedicles. Despite the many differences that may exist among these procedures, they all rely on a few key principles. To make surgery more expeditious and minimize technical challenges, these principles can be incorporated into simple surgical strategies. The authors present a step-by-step approach to help to achieve a successful cosmetic breast reduction for patients with small to moderate breast hypertrophy. Special emphasis is given to intraoperative clues that make this procedure more reproducible and straightforward.

## Abstract 2

Supplemental Digital Content is available in the text.

## INTRODUCTION

The evolution of breast reduction techniques has been a constant reinvention of old ideas.^1^ The objectives of breast reduction are to remove excess parenchyma and skin, to relocate the nipple areola complex (NAC), and to restore a pleasing shape, ideally preserving sensation and reducing scar length.^2,3^

During the last 2 decades, we have experienced a shift toward using skin incision patterns that are best suited to the individual patient and also an increasing use of medial pedicles.^4–9^ Nevertheless, the classic inverted-T, inferior pedicle design with Wise pattern skin markings, remains the most commonly used technique in North America.^10^

Certain intraoperative strategies along with adequate preoperative markings can thoroughly help to simplify a cosmetic breast reduction and achieve satisfactory results, especially for trainees or surgeons at the early days of their practice. This article describes a step-by-step approach to help to achieve a successful cosmetic breast reduction. Most commonly, these steps are applied using a mosque dome skin marking pattern and a superomedial pedicle with vertical and horizontal skin excision, as will be described in this article (Table 1).

**Table 1.**
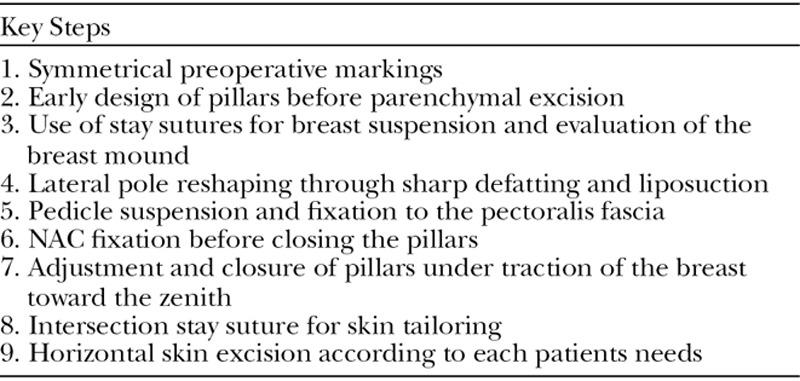
Stepwise Approach in Breast Reduction

## STEP-BY-STEP APPROACH TO A SUCCESSFUL BREAST REDUCTION

### Symmetrical Preoperative Markings

**Fig. 1.**
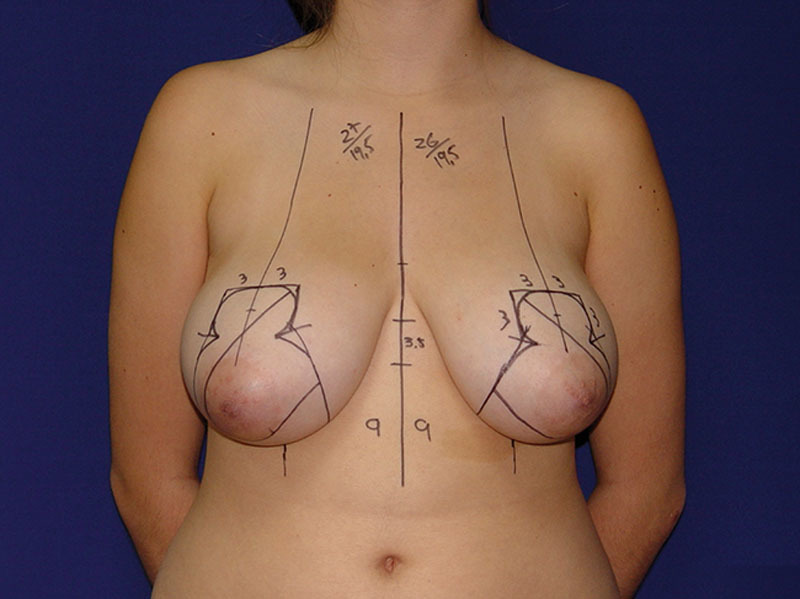
Preoperative breast markings. It is easier to trace the breast meridian by placing a tape measure around the patient’s neck, while the patient keeps a standing position. When using a mosque dome skin pattern, we take advantage of an “assistant square box.” A 3 × 3 cm incomplete square is drawn extending at both sides from the breast meridian. Then the dome is drawn freehand by uniting the mid-point of the inner sides of the square. The pedicle base width is outlined by tracing an oblique line which starts in the superior medial two thirds of the dome, then goes caudally leaving a 1 cm margin of skin surrounding the areola and ends around the mid-third of the medial vertical marking, keeping a base width of 6–8 cm.

The desired breast meridian should be outlined following the ideal position of the NAC on the breast mound. This is done irrespective of what the actual location of the nipple is. Medial markings must be symmetrical between both breasts (Fig. 1). This is of upmost importance for any skin marking pattern because the medial extent of excision will mostly influence the symmetry of the breast shape and scar location. In highly asymmetric breasts, lateral skin markings may differ between both sides, in order to adjust the degree of lateral parenchymal excision.

The center of the new nipple position is estimated by tracing a horizontal line 1 cm over the inframammary fold (IMF) transposition and then marked on the breast mound at the level of the breast meridian. The superior border of the areola is outlined approximately 2 cm over this marking. This can be adjusted according to the patient’s height. The areola diameter is standardly set at 4 cm (**see** video, Supplemental Digital Content 1, which displays a detailed explanation of the marking procedure and steps outlined in this article. This video is available in the “Related Videos” section of the Full-Text article at PRSGlobalOpen.com or available at http://links.lww.com/PRSGO/A988).

**Video Graphic 1.**
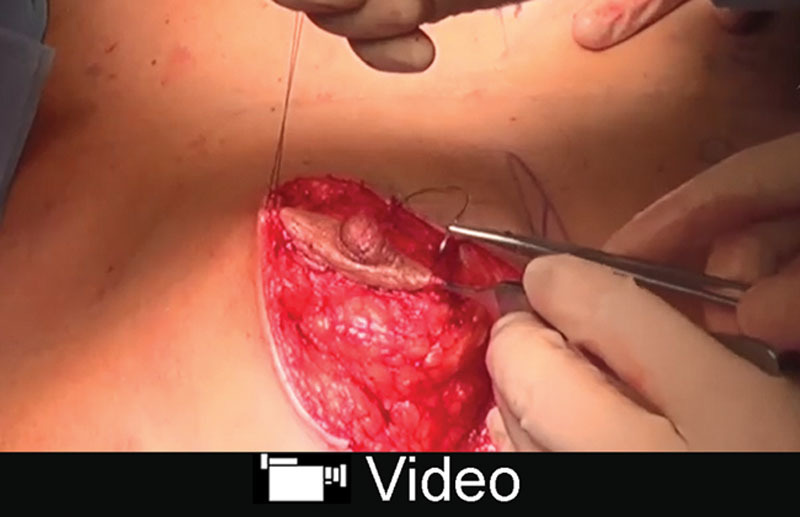
See video, Supplemental Digital Content 1, which displays a detailed explanation of the marking procedure and the steps outlined in this article. This video is available in the “Related Videos” section of the Full-Text article at PRSGlobalOpen.com or available at http://links.lww.com/PRSGO/A988.

### Early Design of Pillars

Before completing the parenchymal excision, the medial and lateral pillars limits are promptly defined. Two vertical lines measuring 7 cm are traced caudally on each side, extending from the lower limit of the new areolar opening. This will correspond to the pillars length. Clear definition of the pillars extension helps to keep flaps of adequate thickness during tissue excision and also configure the breast base. The medial pillar is kept full thickness to improve medial fullness and breast projection. The lateral pillar is thinned up to around 2 cm. Parenchyma is excised at the inferior and lateral areas following the mastectomy cleavage plane leaving an adipocutaneous flap of 4–5 mm.

### Key Untied Stay Sutures

Two nylon 3-0 stay sutures are placed between the new areolar opening and the areola. These sutures are left untied and held in place with 2 Kelly forceps. They must be placed after parenchymal excision has been completed. Stay sutures are key because they offer several advantages at this stage: traction of the sutures toward the zenith gives a 3-dimensional perspective of the new breast, it favors size estimation between both breasts and helps during pillars closure.

### Restoring Lateral Breast Contour

After the lateral pillar has been shaped, we perform a curvilinear excision of glandular tissue at its caudal limit. This helps to restore the natural lateral breast contour. The extent and location of the excision are estimated by outlining on the skin what the ideal lateral breast curvature would be. Parenchyma is then excised leaving a thin dermal flap. In large reductions, we also add liposuction of the lateral breast as an adjunct for improving shape, thus avoiding the need to have extended horizontal scars. This is done at the end of the procedure once the complete breast has already been reshaped.

### Pedicle Suspension

According to the type of pedicle, this should be rotated or displaced superiorly, but under no tension. The pedicle is fixated to the pectoralis fascia in the superior limit of the upper pole dissection, using 2–3 stitches of nonabsorbable sutures. The limit of the cephalic dissection should be kept around the most superior extent of the breast footprint.

### NAC Fixation Before Pillars Closure

The NAC is sutured to the new areolar opening before closing the pillars. This prevents from undesired downward traction of the nipple, reduces tension during closure and avoids abnormal protrusion of the areola after breast assembly.

### Pillars Closure with a Hanging Breast

While the assistant keeps traction of the breast mound toward the zenith, pillars are closed using 2 layers of nonabsorbable sutures. Hanging the breast exposes the correct alignment of the lateral and medial pillars. This makes breast closure easier and usually traduces on an increased projection with better shape.

### Intersection Suture

A stay suture is placed at the intersection between the meridian at the level of the IMF and both medial and lateral flaps. This suture helps during tailoring of skin excess and will be kept in place until breast closure is completed.

### Horizontal Skin Excision

**Fig. 2.**
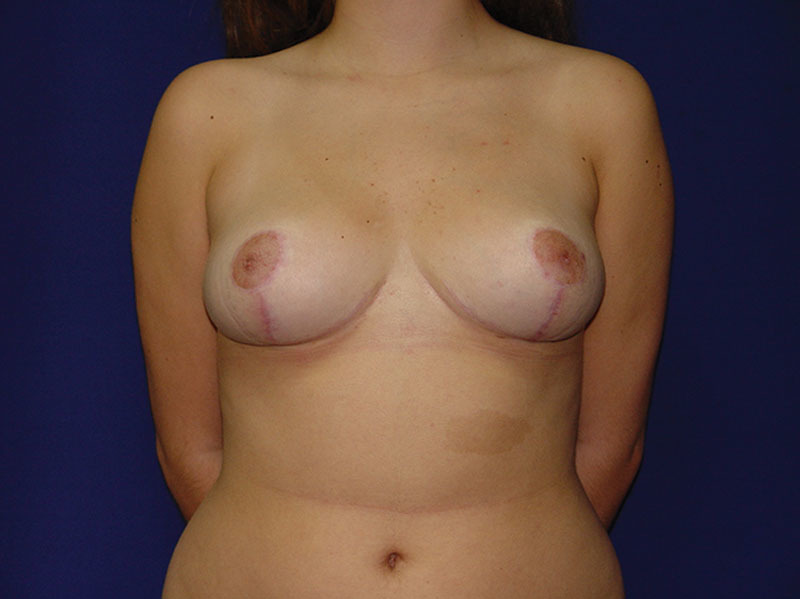
Postoperative, anterior view at 6-month follow-up (same patient shown in Fig. 1 and video). Weight of resected breast was 580 g on the right side, 520 g on the left side, and 50 mL of lipoaspirate from the lateral breast on each side.

Adding a horizontal scar usually improves shape without impairing the patients’ expectations. Horizontal skin excess is excised at the end of the procedure as needed, reducing the final scar length in comparison to skin excision following a default skin pattern (Fig. 2).

Redundant skin is incised at its superior border following a curved shape of upper concavity, which creates 2 inferiorly based dermal flaps. The assistant then pushes the breast caudally so that the natural crease of the IMF becomes evident through the skin excess, indicating the amount of excision needed.

## SUMMARY

**Fig. 3.**
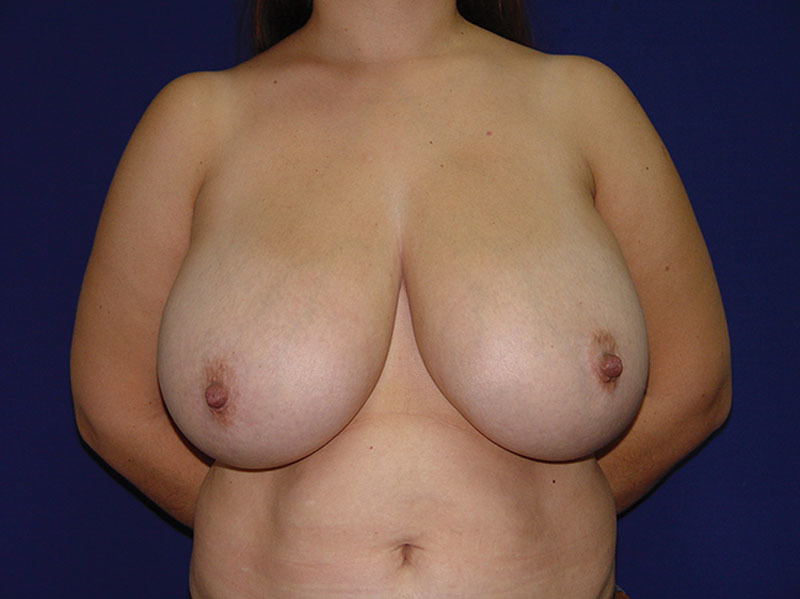
Preoperative, anterior view of 38-year-old woman with moderate bilateral breast hypertrophy.

**Fig. 4.**
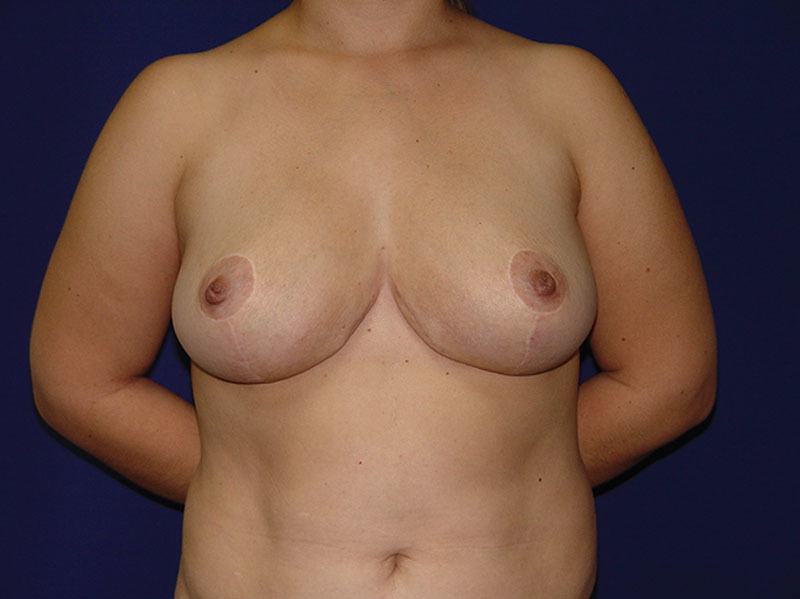
Postoperative, anterior view at 8-month follow-up. Weight of resected breast was 800 g on each side and 400 mL of lipoaspirate from the lateral breast on each side.

In this article, we detail a sequence of clues that simplify the treatment of patients which undergo a cosmetic breast reduction. We usually apply this stepwise approach while using a mosque dome skin marking and a superomedial pedicle, as illustrated in this article. In our experience, this technique is best suited for patients with small to moderate breast hypertrophy including resected volumes below 1,000 g per side (Figs. 3 and 4).

Most of the described steps can be also incorporated when using an inferior pedicle with a Wise pattern marking. If markings are symmetrical, the rest of the steps including treatment of the pillars, use of stay sutures, shaping of the lateral breast, and even skin tailoring remain essentially the same.

This stepwise approach to a cosmetic breast reduction may be a useful adjunct for the successful treatment of patients with small to moderate breast hypertrophy. We believe that this strategy might be especially useful for trainees or plastic surgeons in the early days of their practice.
